# Gene Flow in Genetically Modified Wheat

**DOI:** 10.1371/journal.pone.0029730

**Published:** 2011-12-27

**Authors:** Silvan Rieben, Olena Kalinina, Bernhard Schmid, Simon L. Zeller

**Affiliations:** Institute of Evolutionary Biology and Environmental Studies, University of Zurich, Zurich, Switzerland; University of Melbourne, Australia

## Abstract

Understanding gene flow in genetically modified (GM) crops is critical to answering questions regarding risk-assessment and the coexistence of GM and non-GM crops. In two field experiments, we tested whether rates of cross-pollination differed between GM and non-GM lines of the predominantly self-pollinating wheat *Triticum aestivum*. In the first experiment, outcrossing was studied within the field by planting “phytometers” of one line into stands of another line. In the second experiment, outcrossing was studied over distances of 0.5–2.5 m from a central patch of pollen donors to adjacent patches of pollen recipients. Cross-pollination and outcrossing was detected when offspring of a pollen recipient without a particular transgene contained this transgene in heterozygous condition. The GM lines had been produced from the varieties Bobwhite or Frisal and contained *Pm3b* or *chitinase*/*glucanase* transgenes, respectively, in homozygous condition. These transgenes increase plant resistance against pathogenic fungi. Although the overall outcrossing rate in the first experiment was only 3.4%, Bobwhite GM lines containing the *Pm3b* transgene were six times more likely than non-GM control lines to produce outcrossed offspring. There was additional variation in outcrossing rate among the four GM-lines, presumably due to the different transgene insertion events. Among the pollen donors, the Frisal GM line expressing a *chitinase* transgene caused more outcrossing than the GM line expressing both a *chitinase* and a *glucanase* transgene. In the second experiment, outcrossing after cross-pollination declined from 0.7–0.03% over the test distances of 0.5–2.5 m. Our results suggest that pollen-mediated gene flow between GM and non-GM wheat might only be a concern if it occurs within fields, e.g. due to seed contamination. Methodologically our study demonstrates that outcrossing rates between transgenic and other lines within crops can be assessed using a phytometer approach and that gene-flow distances can be efficiently estimated with population-level PCR analyses.

## Introduction

The frequent use of genetically modified (GM) plants in agriculture demands in-depth ecological risk assessment [1]–[6]. A possible consequence of the release of GM crops can be unintended gene flow to non-GM conspecifics or to wild relatives [7]–[12]. Gene flow can increase the ability of a population to respond to a changing environment due to increased genetic diversity [13]. In plants, gene flow occurs not only by migrating individuals (seed dispersal) but also by migrating gametes, i.e. pollen dispersal. Gene flow via pollen dispersal can occur within and between populations and occasionally even between species [14], [15]. Understanding this process is critical to ensuring the coexistence without gene exchange of GM and non-GM crops [16], [17]. In particular, data about pollen-mediated gene flow are essential to establish appropriate isolation distances between the two [18]. In practice, isolation distances should be large enough to achieve the European Union (EU) GM-adventitious-presence-labeling threshold for food and feed, which allows a maximum contamination of 0.9% GM material in non-GM produce [19].

Previous studies about gene flow in conventional wheat, a predominantly self-pollinating species [20], have found cross-pollination rates of 1–2% for plants in close proximity [13], [21]–[23], which rapidly decreases with greater distance between pollen donor and pollen recipient [20], [24]. However, Lawrie et al. found that cross-pollination rates, using direct spike contact inside glassine bags, could exceed 10% [25]. There are several reasons why wheat has a low cross-pollination rate compared to other grain species. First, fertilization usually occurs before the florets open, which makes pollination with foreign pollen unlikely. Second, although wheat is a wind-pollinated species [26], its pollen is relatively heavy and settles quickly compared to other grass species [20]. Despite the low rates of gene flow, a maximum cross-pollination distance of 2.75 km has been reported in the literature [27].

While there are numerous studies about gene flow over certain distances, gene flow within stands of crop plants, including wheat, has rarely been analyzed. Such studies are necessary to assess the potential dispersal of GM traits if GM plants occur as contamination within fields planted with non-GM crops, due to contaminated seed material or volunteer seedlings [28]. It is usually assumed that GM-wheat would behave similar to conventional varieties, but only scant evidence corroborates this standpoint [24].

In the present study we used GM and non-GM lines of spring wheat *Triticum aestivum* L. with transgenes conferring resistance against fungal pathogens as a model system to assess gene flow by cross-pollination within stands and over short distances in two field experiments. To assess gene flow within the field, we planted seedlings of four independently transformed *Pm3b* and corresponding non-GM control lines as “phytometers” [29], [30] into plots with four different wheat varieties (experiment 1). The low density of phytometer relative to other plants ensured a high “cross-pollination pressure” from the latter and mimicked a situation of the presence of adventitious GM plants in a non-GM background. Outcrossing events were identified by the hybrid phenotype of plants raised from the seeds produced by phytometer plants. To assess gene flow over short distances, we grew 2.5 m strips of non-GM control lines east and west of 1×1 m GM wheat plots. In this second experiment we determined the cross-pollination rate by pooling offspring seeds from the control lines and testing them for the presence or absence of resistance genes using population-level molecular analyses.

The aims of the study were to (i) measure gene flow within the field from two GM and two non-GM lines planted as pollen-donor backgrounds to four different pairs of GM/non-GM sister lines planted as pollen-recipient phytometers, (ii) to measure the influence of distance between GM pollen donor and non-GM pollen recipient on the cross-pollination rates of three pairs of GM/non-GM sister lines and (iii) to analyze line-specific differences in rates of cross-pollination.

## Materials and Methods

### Genetically modified wheat

We used six GM lines of spring wheat either derived from the Mexican variety Bobwhite SH 98 26 or the Swiss variety Frisal. Four GM lines from the variety Bobwhite SH 98 26 were produced by biolistic transformation in different transformation events and each line carried a single copy of the transgene *Pm3b* [30]. *Pm3b* confers race-specific resistance to powdery mildew and was cloned from hexaploid wheat [31]. The transgene was cloned under the control of the maize *Zea mays* L. ubiquitin promoter [32] and transformants were selected on mannose-containing media using the phosphomannose isomerase (PMI) coding gene as a selectable marker [33]. After regeneration of T0 transformants, four independent T1 families were selected. From each T1 family, an offspring pair was further propagated consisting of a homozygous GM plant (GM lines *Pm3b*#1–4) and a null-segregant, i.e. a plant that inherited neither the *Pm3b* transgene nor the selectable marker (control lines S3b#1–4; [30]).

Two GM lines of the variety Frisal were produced by biolistic transformation using the plasmid MAGUCUM, containing (1) an actin-1 promoter, barley-seed β-1,3-glucanase (*glu*) and CaMV terminator, (2) an ubiquitin-1 promoter, barley-seed chitinase (*chi*), CaMV terminator and (3) the bar gene for selection [34]. The GM line A9 *chi* was positively selected for chitinase expression and the line A13 *chi*/*glu* for chitinase and glucanase expression [35]. The pathogenesis-related proteins chitinase and glucanase are known for their broad antifungal effect and their expression should lead to an increased resistance to powdery mildew [36], [37]. Because for the GM-lines of Frisal we did not have null-segregants, it is conceivable that the differences between GM and non-GM lines in Frisal were not only due to the insertion of the transgene but also to additional events that occurred during transformation, e.g. soma-clonal variation acquired during tissue culture.

For the field experiments we used seeds obtained from the fifth generation of the GM lines *Pm3b*#1–4 and their respective non-GM sister lines S3b#1–4 as controls, and seed obtained from the sixth generation of the GM lines A9 *chi* and A13 *chi*/*glu* and its cultivar Frisal as a control. In addition we used the conventional wheat variety Casana as a further non-GM control line.

### Experiment 1: gene flow within plots

The first part of experiment 1 was a field trial with GM and non-GM wheat lines running from March 2008 until August 2008 at an agricultural research station in Zurich-Reckenholz, Switzerland [30]. Seeds of the variety Frisal, its GM lines A9 *chi* and A13 *chi*/*glu*, and the variety Casana, were sown into eight 1×1.08 m plots per line. These stands acted as pollen-donating wheat backgrounds. In each plot, 400 seeds were sown in six rows with a distance of 18 cm between rows using an Oyjord plot drill system (Wintersteiger AG, Ried, Austria). At the same time, seedlings of the four *Pm3b* lines and the four corresponding control lines (S3b#1–4) were raised individually in the glasshouse and transplanted as “phytometers” [38] into each of the 32 field plots once they showed two or three unfolded leaves. Each of the eight lines was represented by two phytometer plants per plot, resulting in a mixing ratio of 16 transplanted phytometers per 400 sown background plants. With this planting procedure we aimed to maximize chances for pollen transfer from background to phytometer plants. Furthermore, it allowed us to detect outcrossed offspring later on because hybrids between Frisal or Casana and Bobwhite differ morphologically from the parental varieties. The flowering period of background and phytometer plants was continuously recorded. After seed maturation, all phytometer plants were individually harvested and threshed. Seeds originating from a single phytometer mother plant are called seed family in the following text. Four of the eight replicate field plots per background line received fertilizer twice during the growing season, i.e. when the plants unfolded the first leaf and when the flag leaf became visible (each time 3 g N m^–2^ were applied as “Ammonsalpeter 27.5”, Lonza, Visp, Switzerland; see [30] for further details of field design).

The second part of experiment 1 took place from March–August in 2010. We planted offspring seeds of the eight phytometer lines from the field experiment 2008 back to the field site. Only phytometer plants that had flowered at the same time as the corresponding pollen-donating background plants and which produced at least four seeds were used. In total, 146 out of 265 seed families (4 blocks×2 fertilizer treatments x 4 background lines x 8 phytometer lines) met these criteria. A minimum of 4 and a maximum of 16 seeds were planted from each seed family, resulting in a total of 1945 individual offspring. We sowed the seeds in patches of four per seed family into ten plots of 1×4 m by hand. The patches were assigned to positions and plots in a completely randomized design. The positions within a plot formed a grid of three rows with a distance of 18 cm between patches (60 seed patches per plot). The plots were arranged in a grid aligned along an x-axis leading from east to west and a y-axis leading from south to north.The plots were surrounded by additional buffer plants of variety Bobwhite to avoid edge effects on the test plants. Phosphorus and potassium fertilizer had been applied to the plots prior to the seed planting in autumn 2009 at a rate of 46 kg P_2_O_5_ ha^-1^ and 60 kg K_2_O ha^−1^. The amount of mineralized nitrogen, determined at the end of February 2010 in the top 100 cm of the soil was 41.7 kg N ha^−1^. Nitrogen fertilizer was additionally applied immediately after sowing (30 kg N ha^−1^) and another 30 kg N ha^−1^ when the flag leaves of the plants became visible. All plots were sprayed with the herbicide cocktail Concert SX (40% Thifensulfurone, 4% Metusulfurone-methyl; Stähler Suisse AG, Zofingen, Switzerland) on 18 May.

We determined the cross-pollination rate by dividing the number of mature offspring hybrids through the total number of mature plants per phytometer. Hybrids produced by cross-pollination of Bobwhite by Frisal or Casana differed visibly in their morphology from offspring produced by self-pollination or cross-pollination with other Bobwhite plants. They were taller and had a reduced awn length than the parental varieties and suffered from slight hybrid necrosis, which can occur when unrelated wheat varieties are crossed [39]. It should be noted that our cross-pollination rate is equivalent to outcrossing rate, that is, we only counted successful pollination with subsequent seed set and offspring growth as pollination event.

To check the reliability of the morphological assessment of hybrid status, all plant classified as hybrids and 65 randomly chosen plants not classified as hybrids ( =  putatively selfed offspring) were tested for the presence or absence of the transgenes *Pm3b*, *chi* and *chi*/*glu* using Polymerase Chain Reaction (PCR) analysis. DNA was isolated from 200–300 mg of fresh leaf tissue by adapting the method of [40]. For the amplification of the *Pm3b* gene, we chose primer sequences fitting the ubiquitin promoter (5′-ATCTCTGTCGCTGCCTCTGG-3′ and 5′-TGTGCGCTCCGAACAACACG-3′; Sigma-Aldrich GmbH, Buchs, Switzerland). The *chi*/*glu* transgenes were detected by amplification of parts of the bar gene in the MAGUCM plasmid (′5-TCAACCACTACATCGAGACA-3′ and ′5-AGTCCAGCTGCCAGAAAC-3′; Sigma-Aldrich GmbH, Buchs, Switzerland). The amplified DNA was separated and visualized performing gel electrophoresis. In total, 97.5% of the hybrids and the putatively selfed offspring were identified correctly, based on the presence/absence tests of *Pm3b*, *chi*, and *chi*/*glu* transgenes (data not shown). We conclude therefore that the method of hybrid detection by visual phenotyping was appropriate.

### Experiment 2: Short-distance gene flow between adjacent subplots

The second field experiment took place at the same agricultural research station as experiment 1 and lasted from March–August 2009. Three GM lines *Pm3b*#1, *Pm3b*#2 and A9 *chi* and their corresponding non-GM lines S3b#1, S3b#2 and Frisal were grown in three separate 7×1 m cross-pollination plots (Figure S1). Each plot consisted of one subplot (1×1 m) in the center with GM plants as pollen donors and four subplots (0.5×1 m) on two opposing sides with the corresponding non-GM plants as pollen recipients. The opposing sides were in eastern or western direction of the pollen source because the prevailing winds at the field site blow from the west (Figure S1). The distances between central subplot and side subplots were 0–0.5, 0.5–1, 1–1.5 and 2–2.5 m (a subplot also occurred between 1.5–2 m but was not harvested). As there were four replicate blocks x eight subplots with pollen recipients (distance subplots) x three line combinations, the sample size was 32 for each tested line and 96 in total. The distance subplots were sown with an Oyjord plot drill system (Wintersteiger AG, Switzerland) and the central plots with the GM pollen source was sown by hand. Seeding density was 400 seeds m^–2^ and there were six rows spaced 18 cm apart. The cross-pollination plots were at least 2 m apart from each other and the intervals were filled with tall-growing triticale plants acting as a pollen barrier to minimize cross-pollination between plots. Flowering periods of pollen donor and receptor subplots were similar in order to allow cross-pollination. Nitrogen fertilizer was applied one day before sowing (40 kg N ha^−1^) on 25 March and again when the plants had their first leaf unfolded (30 kg N ha^−1^). Phosphorus and potassium fertilizer were applied twice at a rate of 46 kg P_2_O_5_ ha^−1^ and 60 kg K_2_O ha^−1^ when the plants unfolded the first leaf and when the flag leaf became visible. The plots were sprayed with the herbicide cocktail Concert SX (40% Thifensulfurone, 4% Metusulfurone-methyl; Stähler Suisse AG) and Starane super (120 g l^−1^ Bromoxynil, 120 g l^−1^ Ioxynil, 100 g l^−1^ Fluroxypyr-metilheptil-ester; Omya Agro AG, Safenwil, Switzerland) in the beginning of May. The plots were treated twice with the insecticide Karate Zeon (100 g l^−1^ Lambda-Cyhalothrin; Syngenta Agro AG, Dielsdorf, Switzerland) against the wheat stem fly (*Chlorops pumilionis* Bjerk.) in the beginning of May and 2 weeks later.

To measure the cross-pollination (and outcrossing) rate in each distance subplot we used a population-level PCR analysis that detected the transgenes *Pm3b* and A9 *chi* in batches of seeds. A single-seed approach was not feasible due to the low expected cross-pollination rates. The optimal size of seed batches was determined in a pilot study with flour from seed batches of defined numbers of GM and non-GM seeds. PCR amplification of DNA extracted from flour of the different seed batches showed that a single GM seed could be detected reliably in 1∶10, 1∶50, 1∶200 and 1∶500 mixtures of GM:non-GM seeds. Potential outcrosses would be heterozygous and would therefore contain only 50% of the DNA of a homozygous GM seed. Taking this into account, we opted for seed batches of 100 seeds in our experiment 2 (Figure S2).

For the analysis of the cross-pollination rate, we collected 5 batches of 100 seeds per distance subplot and produced flour from each batch (TissueLyser, Qiagen Instruments AG, Hilden, Germany). To avoid DNA contamination between batches, the jars used for the milling were sprayed with DNA-ExitusPlusTM (AppliChem GmbH, Darmstadt, Germany) and incubated at 60°C for 10 min to increase the degradation rate of DNA [41].

DNA was extracted from 20 mg flour per sample adapting the method of [42]. To test the DNA extracts for transgene-presence we used the same PCR protocol as described above. If a sample tested positive, DNA extraction and PCR were repeated. Positive samples were therefore based on at least two independent DNA extractions and PCR reactions ().

### Data analysis

The influence of background and phytometer lines on cross-pollination within the plot, measured as the probability of an individual offspring plant to be a hybrid rather than a putatively selfed offspring (experiment 1) was analyzed using generalized linear models (GLMs) with logit link function and binomial error distribution [43]. To account for potential overdispersion, experimental factors were tested with approximate F-tests derived from analysis of deviance tables (see Table S1; [44]). Experimental factors were block, fertilizer application, phytometer line with the four contrasts *Pm3b* vs. control, *Pm3b*#2 vs. other three *Pm3b* lines, variation among these three *Pm3b* lines and variation among the four control lines, background line with the three contrasts Frisal vs. Casana, Frisal GM vs. Frisal control and Frisal A9 vs. Frisal A13, interactions among these terms and phytometer individual (seed family; "Residual" in Table S1). Plants that did not germinate or died due to pest infestation were excluded from further analysis.

Data from experiment 2, the short-distance gene-flow experiment, were analyzed using GLMs with logit-link function and binomial error distribution (Table S2). The dependent variable was the probability to find a transgene in a batch of 100 seeds. In one model, the experimental factor distance was decomposed into a contrast log (distance) and residual variation between distance classes because cross-pollination rates are likely to decrease logarithmically with increasing distance to the pollen source [45]. To investigate differences between very short (0–0.5 m) and short-distance (0.5–2.5 m) gene flow, we split the dataset and analyzed both subsets separately. The highest possible estimate of cross-pollination rate was calculated by dividing the observed amount of positive batches by the total amount of batches. This makes the highly unlikely assumption that in all positive batches all 100 seeds result from cross-pollination. The lowest possible estimate of cross-pollination rate was calculated by dividing the positive samples by the total amount of samples multiplied by 100. This makes the assumption that in all positive batches only 1 seed out of 100 is the result of cross-pollination. Following the maximum likelihood estimation for binomial data, we calculated the values most likely to have produced the observed results [46]. Our approach provides therefore an estimate and not a direct measure of the amount of gene flow. The estimate for the probability is: p  =  1 – ((n–z)/n)^(1/J)^, with n being the total amount of batches, while z represents the positive batches and J the number of seeds per batch, i.e. 100. All statistical analyses were performed with the statistical software R 2.9.2 [47]. The critical significance level was 0.05 for all analyses.

## Results

### Experiment 1: gene flow within plots

40 out of 1192 mature plants could be identified visually as hybrids indicating that 3.36% of all planted seeds had received pollen from foreign wheat varieties (background). Overall, 14.4% of all mother plants produced at least one hybrid seed and 19.6% of all seeds of such plants were identified as hybrids. 21 out of 40 hybrids were crosses between two GM lines, leading to natural but heterozygous pyramiding of *Pm3b* and *chi* or *chi*/*glu* transgenes.

The identity of the mother line, i.e. phytometer plants, significantly influenced the hybridization rate (Table 1 and Table S1): 7.25% of all *Pm3b* seeds were hybridized, which is 6.2 times as many as for the corresponding non-GM control lines (P<0.001 when tested against seed family as a residual in Table S1). There was also significant variation among the four GM lines which could be explained by a contrast between line *Pm3b*#2, which had fewer hybrids, and the other *Pm3b* lines (P  =  0.018). This difference between lines within the group of GM lines was, however, much smaller than the difference between GM and control lines, which can be seen by comparing the deviances in Table S1 (2.3% vs. 16.1% of total deviance). There was no significant variation among the four non-GM control lines (P  =  0.4), indicating that the events during the transformation and tissue culture process did not cause additional variation among lines.

**Table 1:** Cross-pollination rates (mean ± 1 standard error) of the eight pollen recipient lines (Bobwhite phytometer plants) and the four pollen-donor lines (background plants).

Non-GM recipient lines		GM recipient lines		Donor lines	
S3b#1	1.43±0.08	*Pm3b#1*	6.56±0.83	Frisal	2.67±0.10
S3b#2	0.75±0.03	*Pm3b#2*	0.76±0.26	Casana	3.39±0.50
S3b#3	1.90±0.09	*Pm3b#3*	7.24±0.74	A9	6.16±0.41
S3b#4	0.50±0.02	*Pm3b#4*	8.52±0.76	A13	0.61±0.08
mean	0.55±0.06		5.77±0.65		3.21±0.27

Non-GM recipient control lines (S3b #1–4) had significantly lower cross-pollination rates than GM recipient lines (*Pm3b*#1–4). The GM line *Pm3b*#2 with highest transgene expression and lowest fertility had significantly lower cross-pollination rates than the other recipient GM lines. Frisal and Casana are non-GM wheat varieties; A9 and A13 are GM lines based on the variety Frisal. The GM line A9 pollinated significantly more phytometer plants than did GM line A13. Cross-pollination is defined as number of seeds derived from cross-pollination divided by number of all seeds×100.

The identity of the father line, i.e. background plants, also significantly influenced the hybridization rate (Table 1 and Table S1). Among the Frisal fathers, A9 *chi* pollinated more plants than did A13 *chi*/*glu* (P<0.001 for difference A9/A13). Hybrids with Casana fathers had shorter awns than hybrids with Frisal fathers (P  =  0.013 for difference Casana father/Frisal father). Within fathers of the variety Frisal, plants pollinated by Frisal GM fathers (A9 *chi*, A13 *chi*/*glu*) had shorter awns than when pollinated by Frisal control fathers (P  =  0.02 for difference Frisal A9 and A13 father/Frisal control father).

Finally, there were some significant interactions between mother and father lines (Table S1). The Frisal control fathers pollinated more control mothers than did the Frisal GM fathers, which in turn pollinated preferably GM ( = *Pm3b*) mothers (P  =  0.03 for interaction *Pm3b* vs. control x Frisal GM vs. Frisal control).

### Experiment 2: Short-distance gene flow between adjacent subplots

**Figure 1:**
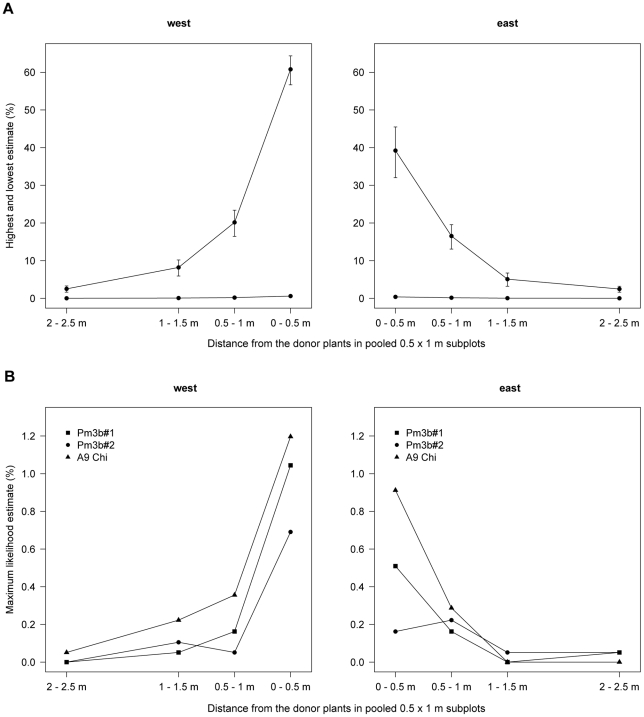
Cross-pollination of GM wheat over short distances and in two wind directions. A: Upper and lower boundaries of cross-pollination rate estimates (mean±1 SE, back-transformed from logit scale) for western and eastern distance subplots. Data from all lines were pooled. B: Maximum likelihood estimate of cross-pollination rate for the western and eastern subplots for the lines *Pm3b*#1, *Pm3b*#2 and A9 *chi*. These estimates indicate cross-pollination rates between 1.2% and 0.16% in the closest and 0.05% and 0.0% in the farthest subplots.

Upper and lower boundaries of the estimated cross-pollination rates are shown in Figure 1A. The upper boundary shows cross-pollination rates assuming that all seeds of a 100-seed sample were genetically modified, if a single seed was tested positive, whereas the lower boundary assumes that only 1 seed in a 100-seed sample was positive. Using the log(distance) model, we found higher cross-pollination probabilities in the west than in the east (P  =  0.048 for difference west/east, Table S2). Furthermore, Frisal A9 *chi* plants were more likely to outcross than Bobwhite plants (P  =  0.02 for difference Bobwhite/Frisal A9 *chi*). We found no significant differences between the two *Pm3b* lines if we combined the data of all distances. However, if we analyzed the subplots closest to the pollen source (0–0.5 m) separately, *Pm3b*#1 was more likely to outcross than *Pm3b*#2 (P  =  0.05 for difference *Pm3b*#1/*Pm3b*#2). Neither varieties, lines nor wind directions differed significantly in subplots further away from the pollen source (0.5–2.5 m).

The actual cross-pollination rates lie between the upper and the lower boundary estimates. We calculated the most likely cross-pollination rate for each distance subplot using a maximum likelihood method (Figure 1B). We found that the estimated overall cross-pollination rate was 0.8% in the west and 0.5% in the east if measured at a distance of 0–0.5 m from the pollen source. Cross-pollination rates decreased more or less linearly with logarithmically increasing distance to the pollen source. Nevertheless, our methods were accurate enough to detect cross-pollination events in 2.5 m distance to the pollen source. The detected rates of 0.05% in the west and 0.02% in the east would be low enough to meet the seed-purity levels set by the European Union [19].

## Discussion

### Increased gene flow to *Pm3b* wheat lines within the field

Large differences among wheat cultivars concerning pollen-mediated gene flow have been reported before and were often attributed to dissimilarities in male fertility and morphological traits [18], [25], [26]. However, we found no other reports showing a higher rate of pollen-mediated gene flow to GM plants than to non-GM plants. Because there were additional differences among the four GM-lines of Bobwhite in our experiment 1, it is conceivable that depending on the insertion event, the particular transgene increased the outcrossing rate to a greater or lesser degree. This would be consistent with phenotypic differences among the four GM lines [30], [48]: lines *Pm3b*#2 and #4 had, measured on other plants but in the same field trial, strongly increased levels of ergot infection, suggesting that their stigmata were exposed for a prolonged period of time which would also have increased their chances to receive foreign pollen [30]. The prolonged exposition of stigmata might in turn have been related to reduced male fertility of the corresponding GM-lines [18]. However, there remains an inconsistency, because line *Pm3b*#2, which had high ergot infection and presumed reduced male fertility, actually had the lowest maternal hybridization rate compared with the other three *Pm3b* lines.

Besides the capacity to receive foreign pollen, the ability to pollinate other plants seems to be important to gene flow as indicated by the differences in pollination rates between father plants from different lines. In this case, however, the difference between Frisal non-GM and GM lines was not significant, whereas the difference between the two GM lines was highly significant. In contrast to the Bobwhite GM lines, we had no null-segregants for the Frisal GM lines. Therefore, it is more difficult to interpret the differences among the pollen donor than among the pollen recipient lines. Finally, the significant interactions between donor and recipient lines in our experiment 1, with a higher crossing success for non-GM x non-GM and GM x GM combinations than for other combinations, hint at the complexity of genetic combining ability between specific lines [49], which demonstrates the importance to test the crossing behavior of GM lines on a case-by-case basis.

We found that on average 3.36% of all tested seeds had resulted from hybridization with neighboring plants. However, this cross-pollination rate varied among the eight wheat lines tested from 0.5–8.5%. These rate measurements are critical to answer questions concerning the EU 0.9% threshold for GM seeds in the harvest [28]. A study with maize *Zea mays* L using a color marker showed an increased percentage of marked seeds at harvest compared to sowing [50]. The contamination percentage at sowing was 1% and on average 2.8 times as high at harvest. The authors therefore concluded that contamination at sowing should be as low as 0.2–0.5% to guarantee the EU 0.9% threshold at harvest. In other words, in the case of maize a seed purity of 0.9% at sowing would not be sufficient to ensure the threshold. However, in the mainly selfing crop wheat, the increase in percentage GM seeds from sowing to harvest would be much smaller even under worst-case scenarios: assuming a cross-pollination rate of 8.5% (the maximum found above) and an initial GM proportion of 0.9%, the proportion at harvest would rise to 1.084% (seeds which are homo- or heterozygous for the transgene). As a caveat we must mention that our phytometer plants occurred at a higher frequency in their plots than would be the expected for adventitious GM plants in a non-GM crop.

### Gene flow in wheat: short and random

The short-distance gene flow estimated in our experiment 2 for wheat matches the results of prior observations in which the average cross-pollination rate was about 1% in close proximity and decreased rapidly with distance from the pollen source [13]. When planning our experiment, we expected to find stronger cross-pollination toward the east than toward the west, due to prevailing winds at the study site. As expected, winds mostly blew from west or northwest during flowering (data not shown). Surprisingly, however, we estimated higher cross-pollination rates in the western subplots. Data from a nearby weather station showed that there were a few hours of easterly or north-easterly winds while 23% of the mother plants were flowering. It might be that cross-pollination occurred mainly during these hours, which then led to a higher cross-pollination in the western subplots. Gene flow also occurred mostly in the opposite direction of prevailing winds in a study by Gatford et al. [24]. We conclude, therefore, that not only prevailing winds are important for cross-pollination, but the winds at the exact time of flowering. Hence, as the time of flowering in wheat is usually short [20], cross-pollination can occur in all directions. This should be considered when planning cross-pollination experiments and determining isolation distances.

We could detect significant differences in gene flow between the varieties Bobwhite and Frisal over a distance of<0.5 m. When comparing the varieties from the subplots which were at least 0.5 m away from the pollen source, no significant differences could be detected anymore. Consistent with the results from experiment 1, *Pm3b*#1 outcrossed significantly more than *Pm3b*#2 up to a distance of 0.5 m from the pollen source. We conclude therefore that the differences between varieties and lines are mainly present over short distances between pollen donor and recipient.

As a methodological corollary, our results from experiment 2 show that pooling can be an appropriate method to gain information on an entire population. Taking population samples of 100 pooled seeds turned out to be the optimal size to estimate rates of cross-pollination over short distances using a maximum-likelihood method. Pooled measures over larger distances or individual measures even over the shortest distance would have led to (too) many negative counts. Choosing the right distance allows not only determination of presence or absence of gene flow, but also an estimation of the quantity of transferred pollen is possible based on probability calculations.

### Conclusions

Our results show that GM lines of wheat can differ in their outcrossing behavior from non-GM control lines. We found that Bobwhite mother plants with a *Pm3b* transgene were more likely to hybridize with other wheat varieties than were non-GM Bobwhite mother plants. This likelihood even varied among the different GM lines. One potential reason for this could have been a more or less prolonged flowering time and stigma exposition among GM lines due to more or less reduced male fertility [18]. We also found that Frisal father plants with a *chi* transgene produced more offspring than Frisal father plants with both *chi* and *glu* transgenes, again demonstrating different outcrossing behavior even among different GM lines. Finally, we could demonstrate that hybrids with two or even three transgenes can occur if different GM plants are planted in close proximity. Such plants could further complicate environmental risk assessments.

Because cross-pollination rates varied strongly between GM and non-GM lines and also among GM lines it may be difficult to develop universal models for pollen-mediated gene flow in wheat. Our results suggest that a case-by-case approach will be required instead [5]. The gene-flow rates which we measured in our experiment 2 indicate that gene flow in wheat mainly occurs over short distances. However, within the field, 14.4% of all maternal plants received pollen from neighboring plants and 3.4% of all offspring seeds were sired by neighboring plants. Each homozygous GM plant is likely to outcross with several neighbors which will result in plants heterozygous for the transgene. The proportion of GM plants within a population is therefore likely to increase. If we take a cross-pollination rate of 3.4% and assume an initial GM contamination of 0.9%, 0.931% of all offspring seeds would contain at least one copy of the transgene. If all plants would have been cross-pollinated, this rate would increase to 1.79% in one generation. We conclude that the determination of cross-pollination rates within the field might be more important than cross-pollination over a distance in order to define appropriate threshold limits necessary to allow coexistence of GM and conventional farming systems.

## Supporting Information

Figure S1 **Schematic design of a cross-pollination plot.**
(PDF)

Figure S2 **PCR analysis from flour of different seed mixtures containing 10%, 2%, 0.5% and 0.2% GM seeds.**
(PDF)

Figure S3 **PCR analysis of wheat flower shows presence or absence of transgenes.**
(PDF)

Table S1 **Factors influencing the rate of cross-pollination of GM and non-GM wheat in experiment 1.**
(PDF)

Table S2 **Factors influencing cross-pollination rates in experiment 2.**
(PDF)
