# Supplementary material for: Gene Flow in Genetically Modified Wheat
Source: PLoS One. 2011 Dec 27;6(12):e29730. doi: 10.1371/journal.pone.0029730 (PMC3246478; doi:10.1371/journal.pone.0029730)

Figure S1. Schematic design of a cross-pollination plot. In the centre a 1 m^2 quadratic subplot of GM wheat was sown as a pollen source. In the eastern and western direction corresponding non-GM plants were sown as pollen recipients into distance subplots (0.5 x 1 m). The lightly shaded distance subplots were harvested after seed maturation.

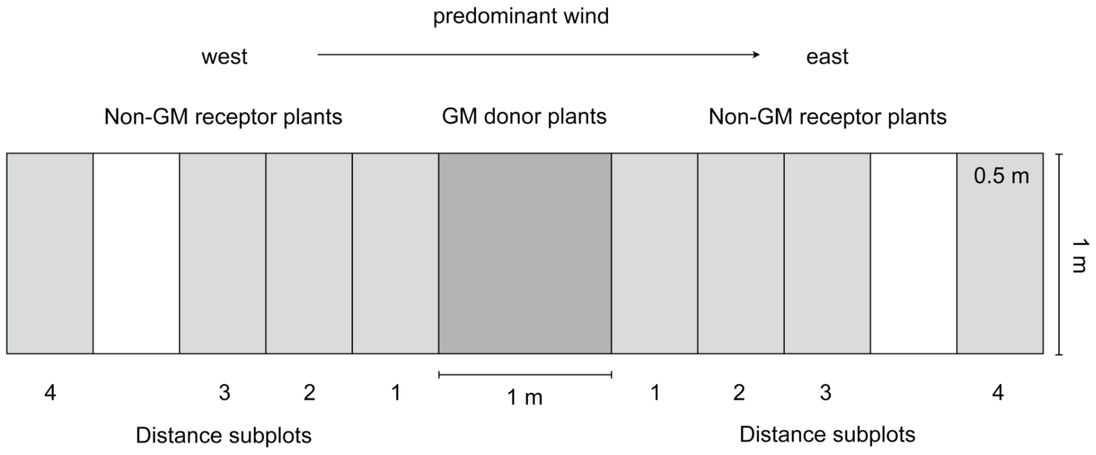

Supplement: Figure S1 — Schematic design of a cross-pollination plot. (PDF) [file pone.0029730.s001.pdf]
