# Supplementary material for: Gene Flow in Genetically Modified Wheat
Source: PLoS One. 2011 Dec 27;6(12):e29730. doi: 10.1371/journal.pone.0029730 (PMC3246478; doi:10.1371/journal.pone.0029730)

Figure S2. PCR analysis from flour of different seed mixtures containing 10%, 2%, 0.5% and 0.2% GM seeds. The positive bands show decreasing signal strength as the proportion of GM seed material decreases. Each analysis was replicated three times.

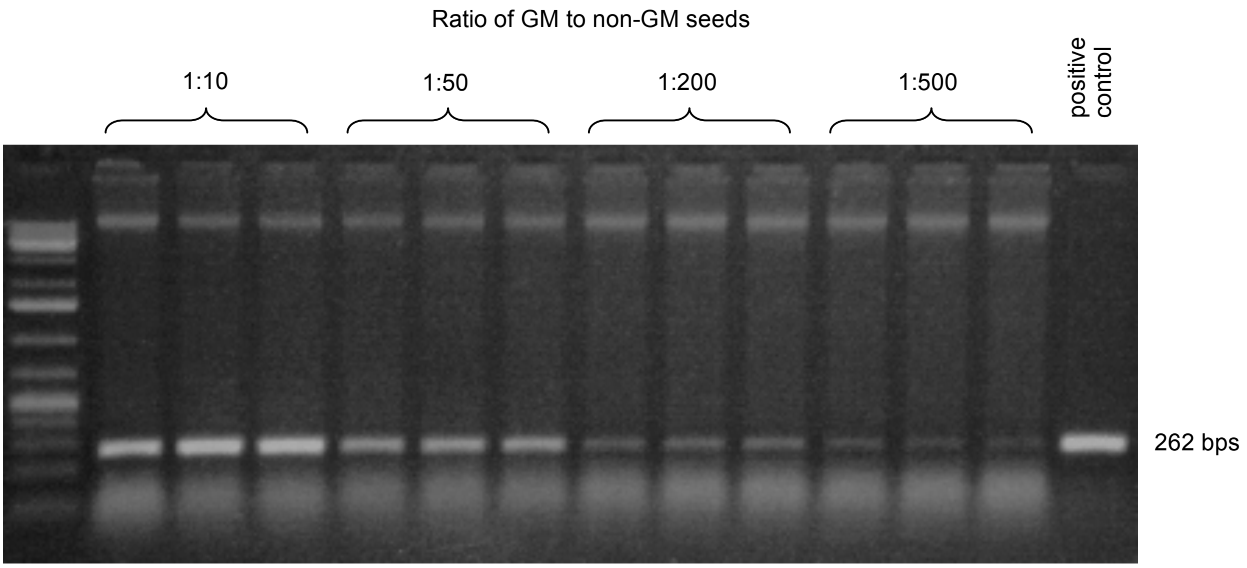

Supplement: Figure S2 — PCR analysis from flour of different seed mixtures containing 10%, 2%, 0.5% and 0.2% GM seeds. (PDF) [file pone.0029730.s002.pdf]
