# Supplementary material for: Gene Flow in Genetically Modified Wheat
Source: PLoS One. 2011 Dec 27;6(12):e29730. doi: 10.1371/journal.pone.0029730 (PMC3246478; doi:10.1371/journal.pone.0029730)

Figure S3. PCR analysis of wheat flower shows presence or absence of transgenes. Distinct white bands at the same height as the positive control indicate successful amplification of transgenic promoter regions (columns nr. 1, 3, 6, 8, 9, 10).

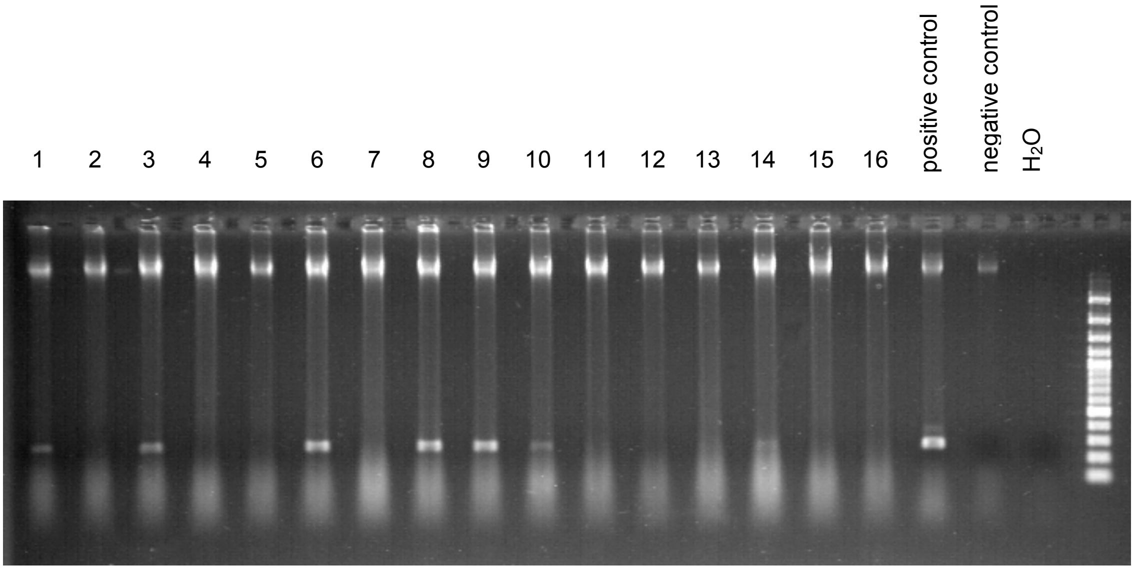

Supplement: Figure S3 — PCR analysis of wheat flower shows presence or absence of transgenes. (PDF) [file pone.0029730.s003.pdf]
