# Supplementary material for: Gene Flow in Genetically Modified Wheat
Source: PLoS One. 2011 Dec 27;6(12):e29730. doi: 10.1371/journal.pone.0029730 (PMC3246478; doi:10.1371/journal.pone.0029730)
Supplement: Table S1 — Factors influencing the rate of cross-pollination of GM and non-GM wheat in experiment 1. (PDF) [file pone.0029730.s004.pdf]

Table S1. Factors influencing the rate of cross-pollination of GM and non-GM wheat in experiment 1. This analysis of deviance table shows the effects of block and fertilizer (conditions under which parental plants grew in the 2008 field experiment), pollen recipient line identity (one contrast between Bobwhite GM and non-GM lines: "*Pm3b* vs. control"; two contrasts within GM lines: "*Pm3b*#2 vs. residual *Pm3b* lines" and "within residual *Pm3b* lines"; residual differences between control lines: "Control"), pollen donor line identity (one contrast between Frisal and Casana: "Frisal vs. Casana"; one contrasts between Frisal GM and non-GM lines: "Frisal GM vs. Frisal control"; one contrast between GM lines: "Frisal A9 vs. Frisal A13") as well as their interaction on the rate of cross-pollination. Abbreviations: df = degree of freedom, % DV = % deviance change due to addition of terms to model, F pr. = error probability based on approximate F-ratios (ratios of mean deviance changes).

Source of variation	df	% DV	F pr.
Block	3	3.1	0.052
Fertiliser application	1	0.2	0.461
Pm3b vs. control	1	16.1	< 0.001
Pm3b#2 vs. residual *Pm3b* lines	1	2.3	0.018
Within residual *Pm3b* lines	2	0.5	0.566
Control	3	1.2	0.400
Frisal vs. Casana	1	0.1	0.566
Frisal GM vs. Frisal control	1	0.0	0.924
Frisal A9 vs. Frisal A13	1	9.6	< 0.001
Pm3b vs. control x Frisal vs. Casana	1	0.0	0.935
Pm3b vs. control x Frisal GM vs. Frisal control	1	1.9	0.030
Pm3b vs. control x Frisal A9 vs. Frisal A13	1	0.2	0.444
Pm3b#2 vs. residual *Pm3b* x Frisal vs. Casana	1	0.3	0.352
Pm3b#2 vs. residual *Pm3b* x Frisal GM vs. Frisal control	1	0.7	0.183
Pm3b#2 vs. residual *Pm3b* x Frisal A9 vs. Frisal A13	1	0.0	0.992
Control x Frisal vs. Casana	3	3.5	0.035
Control x Frisal GM vs. Frisal control	3	1.3	0.365
Control x Frisal A9 vs. Frisal A13	3	0.0	1.000
Within residual x Frisal vs. Casana	2	1.1	0.260
Within residual *Pm3b* x Frisal GM vs. Frisal control	2	7.0	< 0.001
Within residual *Pm3b* x Frisal A9 vs. Frisal A13	2	7.6	< 0.001
Residual	110	43.3	
Total	145	100	
