# Supplementary material for: Gene Flow in Genetically Modified Wheat
Source: PLoS One. 2011 Dec 27;6(12):e29730. doi: 10.1371/journal.pone.0029730 (PMC3246478; doi:10.1371/journal.pone.0029730)
Supplement: Table S2 — Factors influencing cross-pollination rates in experiment 2. (PDF) [file pone.0029730.s005.pdf]

Table S2. Factors influencing cross-pollination rates in experiment 2. This analysis of deviance table shows the effect of the wind direction (west vs. east), distance to pollen source (divided into log(distance) and residuals), plant variety and line identity as well as their interactions on the rate of cross-pollination. Abbreviations: df = degree of freedom, % DV = % deviance change due to addition of terms to model, F pr. = error probability based on approximate F-ratios (ratios of mean deviance changes).

Source of variation	df	% DV	F pr.
Block	3	1.1	0.429
West vs. east	1	1.5	0.048
log(distance)	1	41.4	< 0.001
Residual distance	1	0.0	0.913
Bobwhite vs. Frisal	1	3.7	0.002
Pm3b#1 vs. *Pm3b*#2	1	0.6	0.224
West vs. east x log(distance)	1	0.5	0.281
West vs. east x residual distance	1	0.0	0.768
log(distance) x Bobwhite vs. Frisal	1	0.2	0.531
Residual distance x Bobwhite vs. Frisal	1	0.1	0.640
log(distance) x *Pm3b*#1 vs. *Pm3b*#2	1	0.9	0.127
Residual distance x *Pm3b*#1 vs. *Pm3b*#2	1	0.1	0.656
Residual	81	50.0	
Total	95	100.0	
